# Adolescent Depression Prevalence and Factors: A Jordanian Cross-Sectional Study

**DOI:** 10.7759/cureus.52546

**Published:** 2024-01-19

**Authors:** Layali N Abbasi, Abdullah Hyasat, Ashraqat Alsha’er, Ayah M Al Warawrah, Ghaith Jaser, Motasem Shelbayeh, Samer Al Wahesh, Tareq A Alkhaldi, Tasneem Shakkah

**Affiliations:** 1 Department of Medicine, Al-Balqa Applied University, Salt, JOR

**Keywords:** public psychiatry, health public, child and adolescent mental health, adolescents and children, depression prevention

## Abstract

Objective

There is a great deal of variation in the prevalence of adolescent depression among nations, but overall, it is a severe and common mental illness that is a major contributor to disability on a global scale. This study aimed to determine the prevalence of depression, the severity of its symptoms, and its characteristics among adolescents residing in Amman, Jordan, in 2023.

Method

A cross-sectional design was used to collect descriptive data from 784 adolescents from Jordan, aged between 14 and 17 years. Participants were administered the Patient Health Questionnaire-9 to assess the prevalence and severity of depression as well as measures of socio-demographics and health history. Significance was set at p ≤.05.

Results

The mean age was 15.56 +/- 1.077 with 55% female and 45% male participants, with a prevalence of moderate to severe depression of 32.4%. This study identified four significant predictors of depression: female sex, cigarette smoking, history of mental health problems, and family history of psychiatric disorders.

Conclusion

Our findings highlight the importance of directing prevention and intervention efforts toward adolescents, considering the projected increase in depression rates across the Arab region. This serves as a warning for policymakers and healthcare professionals to prioritize this demographic in their strategies.

## Introduction

Globally, the prevalence of mental health problems in childhood and adolescence ranges from 17% to 20% [1]. It is estimated that one-third to one-fourth of adolescents have mental disorders [2]. Half of all significant adult psychiatric disorders begin by the age of 14 years [3]. However, only a small percentage of these adolescents experience distress or impairment severe enough to warrant intervention [4]. Moreover, research has indicated that developing countries have the highest prevalence rates of such disorders [1,2].

Adolescent depression is a mental health issue that is observed globally with a worldwide prevalence of 6% [5], is a leading cause of impairment and disability [6], and is a key factor in suicidal behavior [7]. According to statistical data, suicide ranks as the third most prevalent cause of mortality among adolescents in both the United States and Europe [8]. Between 2007 and 2018, the frequency of lethal suicide among teenagers and young adults in the United States increased by 57.4% [9]. Depression is significantly associated with other health and developmental issues in adolescents, including lower educational attainment, substance abuse, violence, and poor reproductive and sexual health [10]. According to the American Academy of Child and Adolescent Psychiatry in 2007, adolescents who experience depressive disorders are highly susceptible to legal issues and exposure to unfavorable life events [11]. A considerable percentage of adolescents with depression are also prone to developing other mental disorders, such as substance use, anxiety, or behavioral disorders, as well as chronic illnesses [12].

Until approximately 30 years ago, depression was primarily viewed as an affliction affecting adults. The notion that children are not yet sufficiently developed to experience depressive disorders was widely held, and it was commonly believed that low mood among adolescents was a natural component of typical teenage mood fluctuations [13]. The prevalence of depression increases significantly during the developmental phase from childhood to adolescence, with an estimated increase from 5% during preadolescence to 20% in young adulthood [14].

The identification of risk factors is considered the initial step in early intervention, which has yielded favorable outcomes for adolescents [15]. Sex plays a significant role in the prevalence of depression, with levels of depression increasing more rapidly in girls than in boys during early adolescence [16]. According to a systematic review and meta-analysis of longitudinal studies, certain modifiable factors in adolescence significantly impact the likelihood of developing depression. These factors include the use of substances such as alcohol, tobacco, cannabis, and other illicit drugs [17]. According to the longitudinal BELLA study, the presence of mental health issues in parents, as reported by the parents themselves, was a significant predictor of both self-reported depressive symptoms in their children and adolescents as well as the progression of these symptoms over time [18].

This study aimed to determine the prevalence of depression and its associated risk factors among adolescents in Jordan.

## Materials and methods

A cross-sectional study was conducted in 2023 in Amman, the capital city of Jordan, following the necessary ethical protocols, as evidenced by the signatures of approval obtained from Al-Balqa Applied University and the Ministry of Education (approval number: 3/10/29425).

A multistage sampling methodology was employed to randomly select eight schools. The inclusion criteria encompassed Jordanian adolescents, regardless of sex, whose ages ranged from 14 to 17 years, whose parents provided informed written consent, and the absence of medical conditions.

Two questionnaires were presented to the students and their parents. The first involved data gathering, including sociodemographic information (age, sex, nationality, family income), cigarette use, and a personal or family history of mental illnesses. Owing to cultural sensitivity concerns, the Ministry of Education did not permit any questions that addressed alcohol or drug usage.

The second was the nine-item Patient Health Questionnaire (PHQ-9), a tool deemed diagnostically valid to assess the symptoms of depression [19]. Among adolescents, the PHQ-9 has demonstrated a sensitivity of 89.5% and specificity of 78.8% [20]. The PHQ-9 is a brief, nine-item screening tool that assesses the presence and severity of depressive symptoms. Responses are measured on a four-point Likert-type scale, wherein the responses are aggregated using a scoring range of 0 (not at all) to 3 (almost every day). This approach is commonly employed in academic research to assess the frequency of certain behaviors or attitudes [21]. The scores for each participant ranged from 0 to 27, with higher scores indicating more severe depressive symptoms. Based on this score, depression symptoms were classified as moderate for scores between 10 and 14, moderate to severe for scores between 15 and 19, and severe for scores between 20 and 27 [20]. The psychometric properties of the Arabic version of the PHQ-9 make it an acceptable screening tool for depression among adolescent refugees of Arabic origin [22].

Statistics

SPSS 29.0 (IBM; Armonk, New York, USA) was used to analyze the data. T-tests, analyses of variance, and linear regression analyses were performed on the data. Significance was set at p ≤.05. If only one question was left unanswered, a simple mean imputation was used to handle the missing data. If more than one question remained unanswered, it was deleted.

## Results

In addition to predicting depression levels as a function of participant characteristics, this study sought to determine the prevalence of depressive symptoms among school-age adolescents (14-17 years) in Amman. This study also summarizes school-age adolescents’ demographic variables and presents the statistical analysis techniques employed to address the main study questions. The response rate was 71%, with 784 of the 1,100 participants completing the surveys.

The study enrolled 784 school-age adolescents (mean age = 15.56 ± 1.01 years). Of these, 353 were boys (45.0%) and 431 were girls (55.0%). Most participants (691, 88.1%) reported living with both parents, and 694 participants (88.5%) reported being nonsmokers. Additionally, 758 participants (96.7%) reported never having been seen by a psychiatrist, and 679 participants (86.6%) reported not having any relatives with mental or psychological illnesses. The distribution of monthly household income categories exhibited convergence, although approximately one-third of the sample reported earning between 300-500 Jordanian dinar (JOD) per month. The results are summarized in Table 1.

**Table 1:** Participants’ characteristics (N = 784) JOD: Jordanian dinar The data have been presented as N ( number) and % (percentage). Significance was set at p ≤.05.

Variables	Category	Frequency	Percentage
Sex	Female	431	55.0
	Male	353	45.0
Living with	Both parents	691	88.1
	Father only	33	4.2
	Mother only	60	7.7
Smoking status	Non-smoker	694	88.5
	Smoker	90	11.5
Family monthly income/JD 1 JOD = 1.41 USD	Less than 300	147	18.8
	300-500	236	30.1
	501-700	134	17.1
	701-1000	138	7.6
	More than 1000	129	16.5
Has a mental health problem	No	758	96.7
	Yes	26	3.3
Has a parent with a mental illness	No	679	86.6
	Yes	105	13.5

Prevalence of depressive symptoms levels among adolescents aged 14-17 years

Participants’ depression scores were calculated and classified according to the guidelines provided in the PHQ-9 scale manual to determine the severity of their depressive symptoms. The findings presented in Table 2 indicate that 19.6% and 9.1% of the students exhibited moderately severe and severe depressive symptoms, respectively, with the highest mean depression scores of 16.86 and 22.14.

**Table 2:** Depressive symptoms levels among adolescents aged 14–17 years

Depressive symptoms levels	Frequency	Percentage	Mean	Standard deviation
None–minimal	281	35.8	2.0	1.47
Mild	249	31.8	6.89	1.45
Moderate	154	19.6	11.81	1.41
Moderately severe	71	9.1	16.86	1.38
Severe	29	3.7	22.14	2.46
Total score			7.57	4.72

Predictors of depression score

This study utilized backward multiple linear regression analysis to investigate the significance of depression scores. This approach involved starting with a full set of predictors and subsequently eliminating variables with the highest p-values until a final model, comprising the most significant predictors with p-values below a predetermined threshold, was obtained. This study employed a regression model that included several variables: sex, age, living arrangements, family income, cigarette smoking, history of psychiatric visits, and family history of psychiatric disorders. To account for predictors with more than three levels, such as monthly income and living arrangements, dummy variables were created. Reference categories were established by excluding incomes greater than 1,000 JOD and those living with both parents from the model.

Table 3 displays the results of the final backward multiple linear regression analysis. The results indicated that, on average, female students exhibited a higher level of depression than male students (B = 2.082, p < .001). Furthermore, students who smoked cigarettes displayed higher levels of depression than those who did not smoke cigarettes (B = 3.528, p < .001). Similarly, students who had visited a psychiatrist and those who had relatives with mental illness exhibited higher depression scores than those who had not visited a psychiatrist or those who did not have relatives with mental or psychological illness (B = 4.565 and 2.333, respectively; p < .001).

**Table 3:** Backward multiple linear regression analysis to predict depression scores JOD: Jordanian dinar F(7,776) = 16.286, p < .001, adj. R2 = 12.0% B: unstandardized coefficient

Predictors for depression final model (5)	Unstandardized coefficients		Standard coefficients	t	p
	B	Standard error	Beta		
Sex (female)	2.082	0.395	0.181	5.274	< .001
Smokers	3.528	0.618	0.197	5.709	< .001
Has a mental health problem	4.565	1.091	0.143	4.186	< .001
Has a parent with a mental illness	2.333	0.570	0.139	4.091	< .001
Income less than 300 JOD/month	1.980	0.520	0.135	3.807	< .001
Income (300–500) JOD/month	0.965	0.442	0.077	2.181	.030

Family income level was a significant predictor of depression among students. Specifically, students living in families with low monthly incomes (< 300 or 300-500 JOD) exhibited more depressive symptoms than those living in families earning over 1,000 JOD/month (B = 1.980, p < .001; B = 0.965, p = .030, respectively). The impact of students’ age and living situation on their depression scores was non-significant, as these variables were not included in the final model after five iterations. The significant variables included in the model accounted for an adjusted R-squared value of 12.0%, explaining the variation in depression scores.

## Discussion

Adolescent depression is a widespread and severe psychiatric illness with a global prevalence of 6%.

Our survey found that 64.2% of the adolescents experienced depression at the time of the study. In this sample, 31.8% of participants experienced mild depression, with a 32.4% prevalence of moderate-to-severe depression, of which 3.7% reported having severe depression. These results align with those of a prior study in Jordan in 2017, which reported a 34% prevalence of moderate-to-severe depression [23]. Our results are also consistent with those of a similar study conducted in Saudi Arabia in 2019 using the same screening instrument [24]. However, our results are much higher and inconsistent with those of comparable studies conducted in developed nations, which found a prevalence rate of 4%-8.2% [25]. These variations can be attributed to cultural differences, study populations, data collection instruments, and sampling issues.

A systematic review regarding the global prevalence of depression among adolescents showed that female adolescents have the highest risk of developing depression [26], and according to our study, female participants exhibited a higher prevalence of depressive symptoms than male participants. This finding could be attributed to the post-pubertal period, as advancing puberty has been linked to an increased risk of depressive symptoms in girls [27].

We also found that 11.5% of the participants smoked cigarettes, which was an unexpectedly high percentage among adolescents. According to our survey, cigarette smoking was a predictor of depression in adolescents, and past longitudinal studies have indicated that the relationship between smoking and depression is bidirectional [28].

According to our study, having a personal and family history of mental health problems was significantly associated with depression, which coincides with a study conducted in 2017 in Qatar [29]; in contrast to a study conducted in 2022 in Saudi Arabia, which reported no significant relationship between family history and depression [30]. A total of 48.9% of the participants reported a family monthly income of less than 500 JOD. Our findings indicate that adolescents residing in households with low-income levels exhibited markedly elevated depression scores. This is consistent with a previous study that was conducted in Jordan [23] and other studies showing that individuals with lower socioeconomic status are more likely to experience depression [17].

The strength of this study was that it was conducted with a valid depression screening instrument; however, this study has some limitations. First, it was conducted only in Amman and therefore cannot be generalized to the entire Jordanian adolescent population; future research could be conducted on the entire Jordanian adolescent population. Second, the temporal relationship between the exposure risk factors and the development of the outcome of interest could not be established.

## Conclusions

The prevalence of depression among Jordanian adolescents was 64.2%. Hence, to mitigate the escalating incidence of depression in adolescents, it is imperative that mental health policies expeditiously target these determinants. This can be achieved by scrutinizing existing school-based mental health initiatives and campaigns to ensure consistent implementation and monitoring.
